# Isostructural Inorganic–Organic Piperazine-1,4-diium Chlorido- and Bromidoantimonate(III) Monohydrates: Octahedral Distortions and Hydrogen Bonds

**DOI:** 10.3390/molecules25061361

**Published:** 2020-03-17

**Authors:** Maciej Bujak, Dawid Siodłak

**Affiliations:** Faculty of Chemistry, University of Opole, Oleska 48, 45-052 Opole, Poland; dsiodlak@uni.opole.pl

**Keywords:** inorganic–organic hybrid materials, halogenidoantimonates(III), crystal structure, low temperature, octahedral distortion, Hirshfeld surface analysis, hydrogen bonding

## Abstract

Halogenidoantimonate(III) monohydrates of the (C_4_H_12_N_2_)[SbX_5_]·H_2_O (X = Cl, **1** or Br, **2**) formula, crystallizing in the same monoclinic space group of *P*2_1_/*n*, are isostructural, with an isostructurality index close to 99%. The single crystal X-ray diffraction data do not show any indication of phase transition in cooling these crystals from room temperature to 85 K. Both hybrid crystals are built up from [SbX_6_]^3–^ octahedra that are joined together by a common edge forming isolated bioctahedral [Sb_2_X_10_]^4–^ units, piperazine-1,4-diium (C_4_H_12_N_2_)^2+^ cations and water of crystallization molecules. These structural components are joined together by related but somewhat different O/N/C–H···X and N–H···O hydrogen bonded systems. The evolution of structural parameters, notably the secondary Sb–X bonds along with the associated X/Sb–Sb/X–X/Sb angles and O/N/C–H···X hydrogen bonds, as a function of ligand exchange and temperature, along with their influence on the irregularity of [SbX_6_]^3–^ octahedra, was determined. The comparison of packing features and hydrogen bond parameters, additionally supported by the Hirshfeld surface analysis and data retrieved from the Cambridge Structural Database, demonstrates the hierarchy and importance of hydrogen bond interactions that influence the irregularity of single [SbX_6_]^3–^ units.

## 1. Introduction

The crystal structures of mixed inorganic–organic halogenidoantimonates(III) with organic cations are mainly based upon [SbX_6_]^3–^ and [SbX_5_]^2–^ polyhedra (X = Cl, Br or/and I) forming various types of inorganic substructures, and organic cations that are located between and/or within inorganic frameworks [1,2,3,4]. The isolated as well as involved in more complicated [SbX_6_]^3–^, and [SbX_5_]^2–^ units are usually distorted from regularity showing differences in their Sb–X bond lengths and X–Sb–X angles. The distortions are a consequence of the presence of the 5*s*^2^ lone electron pair of the Sb^III^ atom [5,6,7] and can be described in terms of the valence shell electron pair repulsion (VSEPR) model that is supplemented by the ligand close packing (LCP) model [8,9] and further supported by the electronic distortion approach [10,11]. It was also found that the geometrical irregularities of antimony(III) polyhedra are related to interactions of these single inorganic units with both inorganic and organic components of the structure: (i) inorganic polyhedra may be joined together by bridging X atoms forming polyhedral infinitive or isolated units—primary deformation and (ii) organic cations may be hydrogen bonded to halogen ligands, changing their geometry relative to the central Sb^III^ atom—secondary deformation [12,13,14,15].

Halogenidoantimonates(III) as the perovskite-like hybrid materials are also of interest because of their physicochemical properties such as luminescent, thermochromic and ferroic, particularly since they can be modified relatively easily by the replacement of halogen or organic structural components [3,16,17,18,19,20,21]. However, it is noteworthy that the ferroic properties, in these compounds, have been limited to particular chemical stoichiometries, e.g., R_3_Sb_2_X_9_ (R—organic cation) [3,18,22,23,24,25,26,27,28,29].

Recently, the structures along with the molecular motions study of two piperazine-1,4-diium halogenidoantimonate(III) monohydrates, (C_4_H_12_N_2_)[SbCl_5_]·H_2_O (**1**) and (C_4_H_12_N_2_)[SbBr_5_]·H_2_O (**2**), were reported. In addition, the phase transition behavior of both compounds was investigated [30,31].

We found that the isostructural crystals of **1** and **2** could serve as a convenient system for investigating the structural consequences of the effect of halogen exchange on the polyhedral distortion in the group of antimony(III) compounds, because of their close relationship and simple inorganic substructure. The introduction into the structure of **2** the heavier, more polarizable and less electronegative Br compared to Cl atoms, opens the possibility of the formation of weaker organic–inorganic N/C–H···X hydrogen bonds and thus weakens the secondary octahedral distortion factor. Further, the piperazine-1,4-diium cations due to their tendency to formation of hydrogen bonds and the relatively large spatial dimensions that create a pattern with appropriate cavities for inclusion of water of crystallization molecules [32,33] provide an opportunity to explore the occurrence, hierarchy and competition of N/C–H···X and O–H···X hydrogen bonds on the distortions of inorganic polyhedra. Moreover, the temperature effect, using the same single crystal samples in order to rationalize the contribution of different factors on the structural changes of crystals of **1** and **2**, was studied.

## 2. Results and Discussion

### 2.1. Structures of **1** and **2**

**Figure 1:**
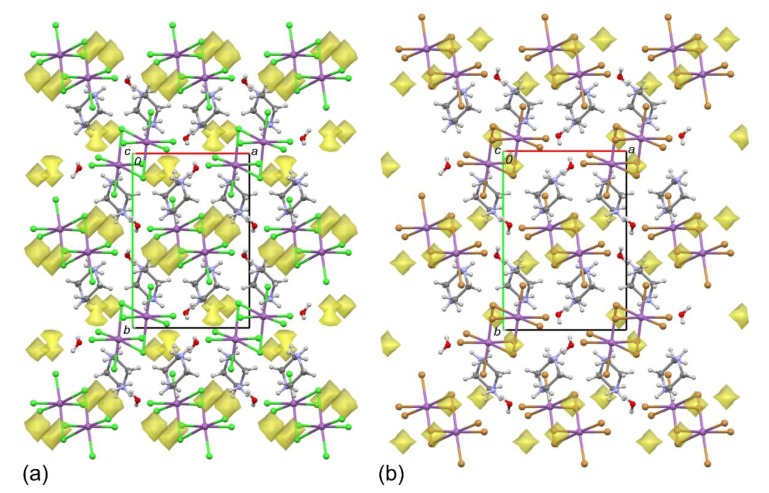
Packing diagrams of **1** (**a**) and **2** (**b**), along the *c*-axes, at 85 K. The intermolecular space accessible to the probing sphere of radius 0.70 Å and the grid spacing of 0.58 Å is indicated in yellow. The void volume is: 1.3%, 16.66 Å^3^ and 0.5%, 7.85 Å^3^ for **1** and **2**, respectively.

The title monohydrates of piperazine-1,4-diium pentachloridoantimonate(III) and pentabromidoantimonate(III) were found to be isostructural—they crystallize in the same monoclinic system with the *P*2_1_/*n* space group and they are characterized by similar unit–cell parameters. Moreover, the positions of corresponding atoms and the crystal packing of both **1** and **2** are similar too (Table 1, Figure 1). The close relationship of these crystals is demonstrated by the calculated unit–cell identity parameters Π of 0.0294 and 0.0313 for the structures determined at 295 and 85 K, respectively, as well as by the very close to 100%, average for 295 and 85 K, isostructurality indexes of 98.8% [34,35,36].

It is also interesting to note that both crystals **1** and **2** show a similar degree of contraction with decreasing temperature from 295 to 85 K. All the *a*, *b* and *c* unit–cell parameters decrease, whereas the monoclinic β angles slightly increase. As a result, the unit–cell volumes at 85 K are 2.8% and 2.3% less than those at 295 K for **1** and **2**, respectively. The structural relationship between **1** and **2** is also manifested in somewhat analogous patterns of voids (the empty spaces in a crystal structure) [37], however the smaller volume of the free spaces and related higher density for **2** should be noted (Table 1, Figure 1).

In both crystals, consisting of the same and fully ordered components, the simple inorganic [Sb_2_X_10_]^4–^ units and organic cations (C_4_H_12_N_2_)^2+^ that are joined together by N/C–H···X hydrogen bonds were found. In addition, the water of crystallization molecules participates in the hydrogen bonded systems, competing for the same X acceptors, through the O–H···X interactions and also as the acceptors for N–H···O hydrogen bonds. Furthermore, the O···O contacts joining the water of crystallization molecules, with an average distance of ca. 2.94 Å, were found. It is noteworthy that the central Sb^III^ atoms occupy general positions and therefore their coordination geometries are not constrained by symmetry. Consequently, the structural changes, induced by halogen atom replacement as well as those occurring by decreasing temperature, are associated with the particular properties of inorganic/organic substructures and changes in the non-covalent interactions. The single crystal X-ray diffraction data do not show any indication of phase transition/symmetry change, in the temperature range 295 to 85 K. This makes it possible to follow continuous changes in the interatomic parameters including those for non-covalent interactions. It was found that temperature does not significantly affect the bond lengths and angles of the organic cations, but it influences the inorganic substructures and the hydrogen bond interactions.

There is one Sb^III^ atom that is surrounded by five X atoms, one piperazine-1,4-diium cation and one water of crystallization molecule in the asymmetric unit of both **1** and **2**. The inorganic substructures are built up from [SbX_6_]^3–^ octahedra connected by one edge forming bioctahedral isolated [Sb_2_X_10_]^4–^ units. Therefore, in the coordination sphere of the central Sb^III^, two X atoms are bridging and four are terminal. At 295 K the shortest are two Sb1–X1/X3 terminal bond lengths, while the longest are located trans to them Sb1–X2^I^/X2 bridging distances. The third pairs of Sb–X involving two terminal mutually trans Sb1–X4/X5 bonds have intermediate lengths. The X–Sb–X angles for X atoms that are located cis to each another clearly deviate from the ideal 90°. In contrast to cis, the trans X–Sb–X angles are somewhat closer to an ideal 180° and are more similar to each other for both crystals (Table 2). The interoctahedral X–Sb–X angles were found to be one of the smallest (82.247(14)° for **1** and 87.723(11)° for **2**) for both studied halogenidoantimonates(III) and other crystals possessing the [Sb_2_X_10_]^4–^ bioctahedral units [37,38,39].

At 85 K, the Sb–X terminal distances involving X1, X3 and X4 are elongated, whereas the longest—both bridging Sb1–X2/X2^I^, as well as terminal Sb1—X5 are shortened in comparison to the corresponding parameters determined at room temperature. The largest changes were noted for bridging Sb1–X2 bonds that are shortened by 0.0237(7) and 0.0210(6) for **1** and **2**, respectively. The X–Sb–X angles are also both smaller and larger than at 295 K. The largest average change of 1.19° of increase and decrease, respectively, with a decreasing temperature was found for the X2–Sb1–X2^I^ and Sb1–X2–Sb1^I^ angles involving the bridging X atoms (Table 2).

The geometry of the bioctahedral [Sb_2_X_10_]^4–^ anions described above in **1** and **2** is comparable to that seen for the same units in other compounds [20,30,31,40]. Moreover, the geometrical parameters of the piperazine-1,4-diium cations in both crystals, adopting the energetically preferred chair conformation [41,42], are consistent with those reported for the structure of piperazine and other analogous compounds containing this cation [30,31,32,43,44,45,46,47,48].

### 2.2. Octahedral Distortions in **1** and **2**

The detailed analysis of the first and the second coordination sphere of the basic inorganic [SbX_6_]^3–^ octahedra and their centrosymmetric bioctahedral [Sb_2_X_10_]^4–^ units together with the environment of organic piperazine-1,4-diium cations in **1** and **2** clarifies the understanding of the interactions in the studied crystals. Furthermore, the comparison to analogous halogenidoantimonates(III) reveals the hierarchy of interactions and elucidate the changes that occur with decreasing temperature and the halogen atom replacement.

As mentioned above, the distortion of polyhedra in the halogenidoantimonate(III) crystals is associated with the presence of the 5*s*^2^ lone electron pair of the Sb^III^ atom and is considered to be a non-bonding effect that occurs for the post-transition metal compounds, in which the central metal shows an oxidation state of two lower than that one resulting from its periodic table group number. It was also found that the elongation of Sb–X bonds is consistent with the structural trans effect [14,49,50,51,52] and is associated with the shortening of oppositely located Sb–X distances.

The comparison of Sb–X bond lengths as well as X–Sb–X angles in **1** and **2** with corresponding geometrical parameters found for the ‘non-distorted’ isolated octahedra (in which all X atoms are engaged in hydrogen bonds) [SbCl_6_]^3–^ [53] and [SbBr_6_]^3–^ [54] demonstrates that the distortion of the single [SbX_6_]^3–^ units is mainly associated with the primary distortion factor, i.e., is the consequence of the formation of [Sb_2_X_10_]^4–^ units. Clearly, while all the Sb–Cl bonds in the ‘non-distorted’ [SbCl_6_]^3–^ octahedron are 2.6552(4) Å [53] the average Sb–Cl distance, in **1**, is 2.7159 and 2.7123 Å at 295 and 85 K, respectively. Only the average bond length of the two terminal trans located ligands of 2.6298 Å at 295 K and the corresponding, slightly shorter distance of 2.6259 Å at 85 K were found to be comparable to the reference distance. A similar behavior is observed for **2**. The similar average Sb–Br distances of two oppositely located terminal bonds are 2.7890 and 2.7889 Å at 295 and 85 K, respectively, and they were found to be the closest to the reference 2.8022(5) Å distance of the ‘non-distorted’ [SbBr_6_]^3–^ octahedron [54]. The cis X–Sb–X angles, in the case of **1** and **2** at 295 and 85 K, for both bridging X atoms are clearly above 90° (average of ca. 96°), whereas the average values of the remaining angles are close to the ideal 90°. The trans angles (average of ca. 176°) also deviate from 180°, whereas the average interoctahedral Sb–X–Sb angle of ca. 84° is evidently smaller than the theoretically expected 90° (Table 2).

**Figure 2:**
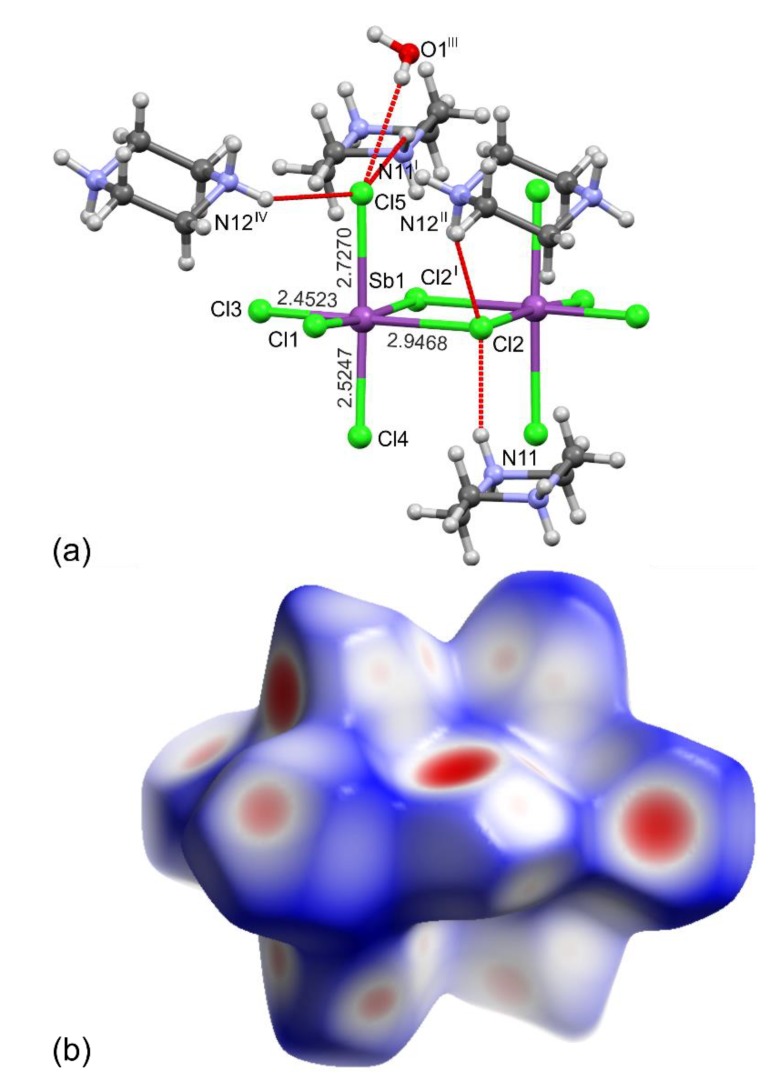
The N/O–H···Cl hydrogen bonds represented by the broken red lines to Cl2 and Cl5 atoms (**a**) along with the corresponding Hirshfeld surface mapped with *d*_norm_ for the [Sb_2_Cl_10_]^4^^−^ bioctahedral unit (**b**) in **1** at 85 K. The overlapping surfaces are marked in red, touching in white, whereas separated in navy blue (**b**). Symmetry codes: (I) –*x* + 1, –*y* + 1, –*z*; (II) *x* – 1/2, –*y* + 3/2, *z* – 1/2; (III) *x* – 1/2, –*y* + 1/2, *z* – 1/2; (IV) –*x* + 1/2, *y* – 1/2, –*z* + 1/2.

The less pronounced secondary distortion results from the differences in geometry and strength of the hydrogen bond interactions between [Sb_2_X_10_]^4–^ units and their environment. The hydrogen bonded to the [Sb_2_X_10_]^4–^ units through N/C–H···X and O–H···X piperazine-1,4-diium cations and water of crystallization molecules, respectively, participate in the distortion of octahedral Sb^III^ coordination, both in terms of differences between equivalent Sb–X bond lengths and X–Sb–X angles. The influence of these non-covalent interactions is seen in the geometrical parameters involving the longest Sb–X distances (Table 2 and Table 3, Figure 2). Comparing Sb–X bond lengths to X4 and X5, which are neither bridging nor opposite to bridging, we found a difference of ca. 0.2 Å for both **1** and **2**, that implies the shift of the electron cloud in the direction of X5. This inequality can be explained by the presence of relatively strong hydrogen bonds in which X5, in contrast to X4, is involved.

The largest change in the D···A distances with cooling was found for O1–H12···X3 interactions that are shortened by 0.063(3) and 0.072(4) Å for **1** and **2**, respectively. The shortening is associated with the elongation of the Sb1–X3 distances by 0.0090(7) for **1** and 0.0157(6) Å for **2**. At the same time, in line with the structural trans effect, the largest shortening with decreasing temperature (by 0.0237(7) and 0.0210(6) Å for **1** and **2**, respectively) was found for the oppositely located Sb1–X2 distances. It is also of interest to note that with a temperature decrease of 210 K, a decreasing of Sb1···Sb1^I^ distances and interoctahedral Sb1–X2–Sb1^I^ angles that are in relation to shortening of N–H···X2 hydrogen bonds is observed (Table 2 and Table 3, Figure 2).

The preferences for the formation of hydrogen bonds to the specific X atoms correspond well to the calculations of apparent residual δ charge carried by a particular halogen atom, which is related to the sum of the bond order *n*, in which the atom is involved (δ = –(1 – Σ*n*); *D*(*n*) = 2.32 – 1.00 log *n* for **1** and *D*(*n*) = 2.46 – 1.10 log *n* for **2**, *D*(*n*)—observed bond length) [55,56]. These calculations show that the largest negative residual charges were found for the longest bridging X2 and X2^I^ as well as for the terminal X5 atoms that are all involved in the N/O–H···X hydrogen bonds belonging to the strongest interactions in the studied **1** and **2** (Table 2, Table 3 and Table 4, Figure 2).

The irregularity of [SbX_6_]^3–^ octahedra can be also described by the bond length Δ ($\Delta_{}=\frac{1}{6}\sum_{i=1}^{6}{\left[\left(R_{i}-\bar{R}\right)/\bar{R}\right]}^{}{}^{2}$, $\bar{R}$—average Sb–X bond length within the octahedron and *R_i_* —individual Sb–X bond length of the octahedron) and the bond angle σ^2^ ($\sigma^{2}=\frac{1}{11}\sum_{i=1}^{12}{\left(\alpha_{i}-90\right)}^{}{}^{2}$, α_i_—individual cis-X–Sb–X bond angle of the octahedron) distortion parameters [57,58]. The distortion parameters for **1** and **2** at 295 and 85 K are displayed in Table 5.

By analysing the Δ and *σ*^2^ parameters, it is clear that in the case of **1** the distortion is revealed by both the bond length and the bond angle parameters. In the case of **2,** both the Δ and *σ*^2^ are lower, confirming the less distorted [SbX_6_]^3–^ units in the crystal. Such behavior corresponding to the energy requirements (i.e., it is easier to move the Cl atoms and change the Sb–Cl and Cl–Sb–Cl parameters than the heavier Br atoms) is further confirmed by the distortion parameters found for the isolated [SbX_6_]^3–^ octahedra [15,53,54,59,60,61]. The observation, in both **1** and **2**, that the linear distortions of the [SbX_6_]^3–^ octahedra decrease and at the same time the angular distortions increase with decreasing temperature, is related to the strengthening of hydrogen bond interactions with decreasing temperature. Furthermore, the changes in bond angles with retaining the bond distances are energetically preferred than the elongation of bond distances for the asymmetrically distributed non-bonding interactions.

**Figure 3:**
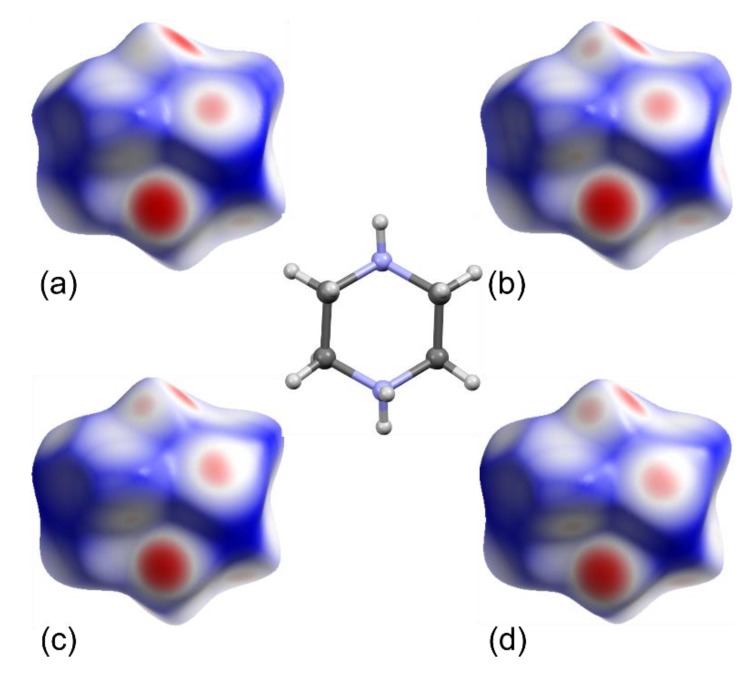
Hirshfeld surfaces mapped with *d*_norm_ for the piperazine-1,4-diium cations in **1** and **2** at 295 (**a**,**c**) and 85 K (**b**,**d**), respectively. The overlapping surfaces are marked in red, touching in white, whereas separated in navy blue.

To further demonstrate, as well as compare and understand the influence of non-covalent interactions, the Hirshfeld surface analysis for **1** and **2** was carried out [62,63,64,65] (Figure 2b and Figure 3). The analysis highlights and additionally proves the aforementioned observations, i.e., there are some slight differences in the O/N/C–H···X hydrogen bonds in **1** and **2** which reflects the strength of the interactions in which the different acceptors of Cl and Br atoms are engaged.

## 3. Materials and Methods

### 3.1. Preparation of **1** and **2**

The commercially available antimony(III) chloride, SbCl_3_ (>99%), antimony(III) oxide, Sb_2_O_3_ (pure), piperazine anhydrous, C_4_H_10_N_2_ (for synthesis), hydrochloric acid, HCl (35%–38%, pure p. a.) and hydrobromic acid, HBr (ACS reagent, 48%) was used, without further purification, for the synthesis and crystallization of (C_4_H_12_N_2_)[SbCl_5_]·H_2_O (**1**) and (C_4_H_12_N_2_)[SbBr_5_]·H_2_O (**2**). 

**1** and **2** were prepared according to a general, previously reported procedure, but the preheated and diluted HCl/HBr acids were used [3,14,30,31]. The molar ratio of starting materials, for both **1** and **2**, was 1:2. C_4_H_10_N_2_ was dissolved in HCl/HBr acid and the resulting solution was used to treat SbCl_3_ and Sb_2_O_3_ solutions in HCl and HBr for **1** and **2**, respectively. The appeared precipitates, in both cases, were dissolved by further addition of the minimum amount of HCl or HBr, stirring and heating up to ca. 325 K. Then the solutions were allowed to stand in a desiccator (P_4_O_10_ was used as a desiccant), at room temperature, forming the single crystals suitable for the X-ray diffraction studies.

### 3.2. X-ray Structure Determination

The intensity data for both **1** and **2**, at 295(2) and 85.0(5) K, were collected on an Xcalibur single crystal diffractometer that was equipped with an Oxford Cryosystems cooler. All the data were subjected to Lorentz, polarization and analytical absorption corrections. The Oxford Diffraction CrysAlis CCD and CrysAlisPro programs were used during the data collection, cell refinement and data reduction [66,67]. SHELX software was used for the structure solutions and refinements [68,69]. The crystal structures were solved by the Patterson method. All hydrogen atoms were located in the subsequent difference Fourier maps, refined restraining to the similar distances (DFIX command of SHELXL [68,69]) for the >CH_2_, >NH_2_ and –OH groups. The hydrogen atom displacement parameters were taken with a coefficient 1.2 and 1.5 times larger than the respective parameters of the carrier carbon/nitrogen and oxygen atoms, respectively. The asymmetric units of the unit cells and the labelling schemes of atoms for both **1** and **2** at 295(2) and 85.0(5) K were chosen to show the structural relationship between the positions of corresponding atoms.

The room-temperature structure of **1** and the structure of **2** at 80(2) K was reported by Sghaier et al. and by Moskwa et al., respectively [30,31], but the low-temperature structure of **1** and room-temperature structure of **2** have not been investigated yet. The present results are basically consistent with the previous reports, Table 1 and Table 2 (cf. Tables S1 and S2), but to avoid biases in the analysis of the structural parameters resulting from comparison of the crystal data obtained for different crystal samples using different instruments, only the data presented in this paper are discussed. Furthermore, in contrast to previous studies, we were able to refine the hydrogen atom positions and discuss, in detail, the octahedral distortions along with the importance of hydrogen bond interactions. Table 3, for the sake of comparison, presents the same set of interactions for **1** and **2** at both the studied temperatures of 295(2) and 85.0(5) K.

The structure drawings were prepared using Mercury [37]. The results of the X-ray diffraction studies were further supported by the Hirshfeld surface analysis provided by CrystalExplorer 2.0 [62,63,64,65] along with the analysis of the data retrieved from the Cambridge Structural Database [37,38,39].

## 4. Conclusions

The structural chemistry of halogenidoantimonates(III) and other technologically important materials, in which central metals show lower oxidation states, is quite intriguing, since the presence of the lone electron pair affects the different degrees of distorted polyhedral coordination. Thus, it is not easy to find the perfectly non-distorted square pyramid or octahedron in the whole group of these compounds. Furthermore, the geometrical parameters within the inorganic substructures of halogenidoantimonates(III) are crucial for their properties, e.g., the Sb–Br bond length variances within [Sb_2_Br_10_]^4–^ influence the thermochromic changes of (C_16_H_20_N_2_)[SbBr_5_] [20].

Two isostructural piperazine-1,4-diium halogenidoantimonate(III) monohydrates (C_4_H_12_N_2_)[SbX_5_]·H_2_O (X = Cl, **1** or Br, **2**) were obtained, in a similar way, from its acidic HCl and HBr solutions, respectively. Both crystals are characterized by an inorganic substructure that is built up from isolated [Sb_2_X_10_]^4–^ bioctahedra, as well as organic piperazine-1,4-diium cations and water of crystallization molecules. It was demonstrated that when two [SbX_6_]^3–^ octahedra are joined together by a common edge forming [Sb_2_X_10_]^4–^ units, the Sb–X bonds show different lengths and the X–Sb–X angles deviate from the ideal 90° and 180°. These deviations are associated with the requirements of the inorganic substructure formation as well as with the strength and distribution of non-covalent interactions.

No phase transition was detected, in both **1** and **2**, within the studied temperature range, i.e., upon cooling from 295 to 85 K. This allows us to monitor the changes occurring, particularly in the case of the longest secondary Sb–X bonds and associated X–Sb–X angles as well as rationalizing the contribution of the hydrogen bonds to the distortion of inorganic polyhedra.

Both studied halogenidoantimonates(III) show unique structural behavior represented by evidently small X–Sb–X interoctahedral angles and related short Sb···Sb separation distances. These parameters are associated with relatively long Sb–X bridging distances and also with the hydrogen bond interactions, in which the bridging X atoms are involved. Such behavior demonstrates the stereochemical activity of the antimony(III) lone electron pair as well as the attractive character of hydrogen bond interactions. The Cl by Br replacement results in less distorted octahedra with regard to bond lengths and bond angles, whereas decreasing temperature leads to the more pronounced changes of bond angles than bond lengths. This could be explained in terms of the size and polarizability of halogen atoms and the energy required to move the X atoms along the Sb–X bonds.

## Figures and Tables

**Table 1:** Selected crystal data for **1** and **2** at 295 and 85 K.

Compound	1	1	2	2
T (K)	295(2)	85.0(5)	295(2)	85.0(5)
Formula	C_4_H_14_Cl_5_N_2_OSb	C_4_H_14_Cl_5_N_2_OSb	C_4_H_14_Br_5_N_2_OSb	C_4_H_14_Br_5_N_2_OSb
*M*_r_	405.18	405.18	627.43	627.43
Crystal system	monoclinic	monoclinic	monoclinic	monoclinic
Space group, *Z*	*P*2_1_/*n*, 4	*P*2_1_/*n*, 4	*P*2_1_/*n*, 4	*P*2_1_/*n*, 4
*a* (Å)	9.54078(13)	9.45434(13)	9.9162(3)	9.82225(15)
*b* (Å)	14.14260(19)	14.05399(19)	14.4090(4)	14.34740(19)
*c* (Å)	10.03963(14)	9.91376(13)	10.3898(3)	10.29729(16)
β (°)	99.1119(13)	99.2085(13)	99.432(3)	99.7664(15)
*V* (Å^3^)	1337.57(3)	1300.28(3)	1464.45(7)	1430.10(4)
ρ_calc_ (g/cm^3^)	2.012	2.070	2.846	2.914
*R_1_, I* > 2*σ*(*I*)	0.0212	0.0140	0.0252	0.0183
*wR_2_*, all data	0.0525	0.0327	0.0500	0.0368

**Table 2:** Selected bond lengths (Å) and angles (°) for **1** (X = Cl) and **2** (X = Br) at 295 and 85 K.

Compound	1	1	2	2
T (K)	295	85	295	85
Sb1–X1	2.3914(5)	2.3936(4)	2.5687(5)	2.5732(3)
Sb1–X2	2.9705(6)	2.9468(4)	3.0884(5)	3.0674(3)
Sb1–X2^I^	3.2308(6)	3.2291(4)	3.2537(5)	3.2528(3)
Sb1–X3	2.4433(6)	2.4523(4)	2.6090(5)	2.6247(3)
Sb1–X4	2.5246(6)	2.5247(4)	2.7049(5)	2.7080(3)
Sb1–X5	2.7350(6)	2.7270(4)	2.8731(5)	2.8697(3)
Sb1···Sb1^I^	4.0832(2)	4.0099(2)	4.3962(5)	4.3428(3)
X1–Sb1–X2	85.66(2)	85.073(12)	87.479(14)	87.133(9)
X1–Sb1–X2^I^	175.28(2)	174.247(12)	176.077(16)	175.030(11)
X1–Sb1–X3	91.13(3)	91.066(14)	92.652(17)	92.467(11)
X1–Sb1–X4	90.50(2)	90.290(13)	91.154(15)	91.122(10)
X1–Sb1–X5	85.66(2)	84.948(12)	86.920(14)	86.227(10)
X2–Sb1–X2^I^	97.753(14)	99.165(9)	92.277(11)	93.245(8)
X2–Sb1–X3	176.70(2)	176.075(12)	177.960(15)	177.964(11)
X2–Sb1–X4	89.783(15)	89.840(11)	90.061(12)	90.183(9)
X2–Sb1–X5	89.695(15)	89.388(10)	87.614(12)	87.392(8)
X2^I^–Sb1–X3	85.42(2)	84.640(12)	87.453(14)	86.981(9)
X2^I^–Sb1–X4	92.771(17)	93.596(11)	92.762(12)	93.832(9)
X2^I^–Sb1–X5	91.083(15)	91.180(10)	89.157(12)	88.839(8)
X3–Sb1–X4	90.981(18)	90.886(12)	91.971(14)	91.820(10)
X3–Sb1–X5	89.329(18)	89.570(12)	90.360(14)	90.591(10)
X4–Sb1–X5	176.147(17)	175.224(12)	177.043(13)	176.492(10)
Sb1–X2–Sb1^I^	82.247(14)	80.835(9)	87.723(11)	86.755(8)

Symmetry code: (I) –*x* + 1, –*y* + 1, –*z*.

**Table 3:** Hydrogen bonds geometries (Å, °) for **1** and **2** at 295 and 85 K.

Atoms	D–H	H∙∙∙A	D∙∙∙A	D–H∙∙∙A
**1**, 295 K				
O1–H11···Cl1^I^	0.85(1)	2.95(3)	3.449(2)	119(3)
O1–H12···Cl3	0.85(1)	2.71(3)	3.414(3)	140(4)
O1–H11···Cl5^II^	0.85(1)	2.70(2)	3.399(3)	141(3)
N11–H112···Cl2	0.89(1)	2.35(2)	3.201(2)	163(2)
N11–H111···Cl5^III^	0.89(1)	2.76(2)	3.3444(19)	125(2)
N12–H122···Cl2^IV^	0.90(1)	2.38(2)	3.256(2)	165(2)
N12–H121···Cl5^V^	0.89(1)	2.51(2)	3.2606(19)	143(2)
C13–H132···Cl4	0.96(1)	2.90(2)	3.555(2)	126(2)
C14–H141···Cl3^V^	0.97(1)	2.79(2)	3.659(2)	150(2)
C14–H142···Cl3^VI^	0.96(1)	2.84(2)	3.675(3)	146(2)
N11–H111···O1^VII^	0.89(1)	2.19(2)	2.943(3)	143(2)
**1**, 85 K				
O1–H11···Cl1^I^	0.85(1)	2.80(2)	3.4198(14)	131(2)
O1–H12···Cl3	0.85(1)	2.57(1)	3.3512(15)	155(2)
O1–H11···Cl5^II^	0.85(1)	2.67(2)	3.3493(14)	138(2)
N11–H112···Cl2	0.89(1)	2.34(1)	3.1859(14)	160(2)
N11–H111···Cl5^III^	0.90(1)	2.76(2)	3.3024(14)	121(1)
N12–H122···Cl2^IV^	0.90(1)	2.38(1)	3.2316(14)	158(2)
N12–H121···Cl5^V^	0.89(1)	2.49(1)	3.2287(13)	140(2)
C13–H132···Cl4	0.96(1)	2.86(2)	3.5277(15)	128(1)
C14–H141···Cl3^V^	0.96(1)	2.77(1)	3.6256(16)	148(1)
C14–H142···Cl3^VI^	0.96(1)	2.80(1)	3.6358(16)	145(1)
N11–H111···O1^VII^	0.90(1)	2.09(1)	2.8926(19)	148(2)
**2**, 295 K				
O1–H11···Br1^I^	0.85(1)	2.92(3)	3.592(4)	137(4)
O1–H12···Br3	0.85(1)	2.67(2)	3.505(4)	169(4)
O1–H11···Br5^II^	0.85(1)	3.07(2)	3.543(4)	117(2)
N11–H112···Br2	0.90(1)	2.56(2)	3.374(4)	151(3)
N11–H111···Br5^III^	0.90(1)	2.90(3)	3.480(3)	124(3)
N12–H122···Br2^IV^	0.90(1)	2.61(2)	3.473(3)	159(3)
N12–H121···Br5^V^	0.90(1)	2.78(3)	3.441(3)	132(3)
C13–H132···Br4	0.97(1)	2.94(3)	3.685(4)	134(3)
C14–H141···Br3^V^	0.97(1)	2.87(2)	3.779(4)	157(3)
C14–H142···Br3^VI^	0.97(1)	2.95(2)	3.787(4)	146(3)
N11–H111···O1^VII^	0.90(1)	2.17(3)	2.939(5)	142(3)
**2**, 85 K				
O1–H11···Br1^I^	0.85(1)	2.93(2)	3.551(2)	132(3)
O1–H12···Br3	0.85(1)	2.61(2)	3.433(2)	165(4)
O1–H11···Br5^II^	0.85(1)	2.99(2)	3.502(3)	121(2)
N11–H112···Br2	0.90(1)	2.51(2)	3.358(2)	157(3)
N11–H111···Br5^III^	0.90(1)	2.96(3)	3.443(2)	115(2)
N12–H122···Br2^IV^	0.91(1)	2.58(2)	3.437(2)	158(3)
N12–H121···Br5^V^	0.90(1)	2.75(2)	3.410(2)	131(2)
C13–H132···Br4	0.98(1)	2.93(2)	3.657(3)	133(2)
C14–H141···Br3^V^	0.97(1)	2.87(2)	3.746(3)	152(2)
C14–H142···Br3^VI^	0.97(1)	2.92(2)	3.743(3)	143(2)
N11–H111···O1^VII^	0.90(1)	2.08(2)	2.896(3)	151(3)

Symmetry codes: (I) –*x* + 1/2, *y* – 1/2, –*z* + 1/2; (II) *x* + 1/2, –*y* + 1/2, *z* + 1/2; (III) –*x* + 1, –*y* + 1, –*z*; (IV) *x* + 1/2, –*y* + 3/2, *z* + 1/2; (V) –*x* + 1/2, *y* + 1/2, –*z* + 1/2; (VI) –*x* + 1, –*y* + 1, –*z* + 1; (VII) –*x* + 3/2, *y* + 1/2, –*z* + 1/2.

**Table 4:** Residual δ charges on the X atoms for **1** (X = Cl) and **2** (X = Br) at 295 and 85 K.

Compound	1	1	2	2
Atoms/T (K)	295	85	295	85
X1	–0.15	–0.16	–0.20	–0.21
X2	–0.65	–0.64	–0.54	–0.53
X2^I^	–0.65	–0.64	–0.54	–0.53
X3	–0.25	–0.26	–0.27	–0.29
X4	–0.38	–0.38	–0.40	–0.40
X5	–0.62	–0.61	–0.58	–0.58

Symmetry code: (I) –*x* + 1, –*y* + 1, –*z*.

**Table 5:** Distortion Δ and σ^2^ parameters for [SbX_6_]^3–^ octahedra for **1** (X = Cl) and **2** (X = Br) at 295 and 85 K.

Parameter	Bond Length Distortion, Δ × 10^3^		Bond Angle Distortion, σ^2^	
Compound/T (K)	295	85	295	85
**1**	12.35	11.93	11.88	16.31
**2**	7.77	7.34	4.90	6.91

## Supplementary Materials

Supplementary Materials are available online. Tables S1 and S2. The set of supplementary crystallographic data for **1** and **2**, at both 295(2) and 85.0(5) K, in the CIF format, is available from the Cambridge Crystallographic Data Centre nos. CCDC 1957876–1957879. These data can be obtained free of charge via http://www.ccdc.cam.ac.uk/conts/retrieving.html, or from the Cambridge Crystallographic Data Centre, 12 Union Road, Cambridge CB2 1EZ, UK; fax: +44 1223 336 033; e-mail: deposit@ccdc.cam.ac.uk or http://www.ccdc.cam.ac.uk.
