# Supplementary material for: Isostructural Inorganic–Organic Piperazine-1,4-diium Chlorido- and Bromidoantimonate(III) Monohydrates: Octahedral Distortions and Hydrogen Bonds
Source: Molecules. 2020 Mar 17;25(6):1361. doi: 10.3390/molecules25061361 (PMC7144372; doi:10.3390/molecules25061361)
Supplement: Supplementary file 1 [file molecules-25-01361-s001.pdf]

Supplementary Material

Isostructural Inorganic-Organic Piperazine-1,4-diium Chlorido- and Bromidoantimonate(III) Monohydrates: Octahedral Distortions and Hydrogen Bonds

Maciej Bujak* and Dawid Siodłak

Faculty of Chemistry, University of Opole, Oleska 48, 45-052 Opole, Poland.
**Correspondence: mbujak@uni.opole.pl*

Table S1. Selected crystal data and structure determination summary for **1** and **2** at 295 and 85 K.

**Compound**	**1**	**1**	**2**	**2**
Formula	$C_4H_{14}Cl_5N_2OSb$	$C_4H_{14}Cl_5N_2OSb$	$C_4H_{14}Br_5N_2OSb$	$C_4H_{14}Br_5N_2OSb$
M_r	405.18	405.18	627.43	627.43
Crystal color; habit	colorless; pillar	colorless; pillar	yellow; pillar	yellow; pillar
Crystal size (mm)	0.32 x 0.27 x 0.21	0.32 x 0.27 x 0.21	0.16 x 0.11 x 0.09	0.16 x 0.11 x 0.09
Crystal system	monoclinic	monoclinic	monoclinic	monoclinic
Space group	$P2_1/n$	$P2_1/n$	$P2_1/n$	$P2_1/n$
a (Å)	9.54078(13)	9.45434(13)	9.9162(3)	9.82225(15)
b (Å)	14.14260(19)	14.05399(19)	14.4090(4)	14.34740(19)
c (Å)	10.03963(14)	9.91376(13)	10.3898(3)	10.29729(16)
β (º)	99.1119(13)	99.2085(13)	99.432(3)	99.7664(15)
V (Å^3)	1337.57(3)	1300.28(3)	1464.45(7)	1430.10(4)
Z	4	4	4	4
ρ_{calc} (g/cm^3)	2.012	2.070	2.846	2.914
T (K)	295(2)	85.0(5)	295(2)	85.0(5)
μ (mm^{-1})	3.031	3.118	15.500	15.872
F(000)	784	784	1144	1144
θ (º)	3.09 - 28.50	3.12 - 28.49	2.83 - 28.50	2.84 - 28.48
Index ranges	$-11 \le h \le 12$	$-11 \le h \le 12$	$-12 \le h \le 13$	$-13 \le h \le 11$
	$-17 \le k \le 18$	$-17 \le k \le 18$	$-19 \le k \le 18$	$-19 \le k \le 18$
	$-13 \le l \le 13$	$-13 \le l \le 13$	$-13 \le l \le 13$	$-13 \le l \le 13$
Reflns collected/unique	10351/3320	10151/3239	11519/3667	11183/3573
R_{int}	0.0228	0.0162	0.0243	0.0222
Data [$I > 2\sigma(I)$]	3055	3069	2860	3148
Data/parameters	3320/161	3239/161	3667/160	3573/160
GooF on F^2	1.095	1.105	1.034	1.049
R_1, wR_2; $I > 2\sigma(I)$	0.0212, 0.0516	0.0140, 0.0323	0.0252, 0.0472	0.0183, 0.0357
R_1, wR_2; all data	0.0237, 0.0525	0.0157, 0.0327	0.0406, 0.0500	0.0243, 0.0368
$\Delta\rho_{min}$, $\Delta\rho_{max}$ (e·Å^{-3})	0.649, –0.759	0.446, –0.423	0.832, –0.817	0.637, –0.620
CCDC	1957876	1957877	1957878	1957879

Table S2. Selected bond lengths (Å) and angles (°) for **1** (X = Cl) and **2** (X = Br) at 295 and 85 K.

**Compound**	**1**	**1**	**2**	**2**
T (K)	295	85	295	85
Sb1–X1	2.3914(5)	2.3936(4)	2.5687(5)	2.5732(3)
Sb1–X2	2.9705(6)	2.9468(4)	3.0884(5)	3.0674(3)
Sb1–X2^{I}	3.2308(6)	3.2291(4)	3.2537(5)	3.2528(3)
Sb1–X3	2.4433(6)	2.4523(4)	2.6090(5)	2.6247(3)
Sb1–X4	2.5246(6)	2.5247(4)	2.7049(5)	2.7080(3)
Sb1–X5	2.7350(6)	2.7270(4)	2.8731(5)	2.8697(3)
Sb1···Sb1^{I}	4.0832(2)	4.0099(2)	4.3962(5)	4.3428(3)
X1–Sb1–X2	85.66(2)	85.073(12)	87.479(14)	87.133(9)
X1–Sb1–X2^{I}	175.28(2)	174.247(12)	176.077(16)	175.030(11)
X1–Sb1–X3	91.13(3)	91.066(14)	92.652(17)	92.467(11)
X1–Sb1–X4	90.50(2)	90.290(13)	91.154(15)	91.122(10)
X1–Sb1–X5	85.66(2)	84.948(12)	86.920(14)	86.227(10)
X2–Sb1–X2^{I}	97.753(14)	99.165(9)	92.277(11)	93.245(8)
X2–Sb1–X3	176.70(2)	176.075(12)	177.960(15)	177.964(11)
X2–Sb1–X4	89.783(15)	89.840(11)	90.061(12)	90.183(9)
X2–Sb1–X5	89.695(15)	89.388(10)	87.614(12)	87.392(8)
X2^{I}–Sb1–X3	85.42(2)	84.640(12)	87.453(14)	86.981(9)
X2^{I}–Sb1–X4	92.771(17)	93.596(11)	92.762(12)	93.832(9)
X2^{I}–Sb1–X5	91.083(15)	91.180(10)	89.157(12)	88.839(8)
X3–Sb1–X4	90.981(18)	90.886(12)	91.971(14)	91.820(10)
X3–Sb1–X5	89.329(18)	89.570(12)	90.360(14)	90.591(10)
X4–Sb1–X5	176.147(17)	175.224(12)	177.043(13)	176.492(10)
Sb1–X2–Sb1^{I}	82.247(14)	80.835(9)	87.723(11)	86.755(8)
N11–C13	1.490(3)	1.4917(19)	1.480(5)	1.496(4)
N11–C16	1.490(3)	1.4968(19)	1.489(5)	1.497(4)
N12–C14	1.489(3)	1.4997(19)	1.483(5)	1.495(4)
N12–C15	1.486(3)	1.4939(19)	1.480(5)	1.491(4)
C13–C14	1.503(3)	1.506(2)	1.491(5)	1.502(4)
C15–C16	1.504(3)	1.510(2)	1.504(6)	1.521(4)
N11–C13–C14	110.29(17)	110.54(12)	111.0(3)	110.7(2)
N11–C16–C15	110.79(17)	110.88(12)	111.1(3)	110.9(2)
N12–C14–C13	109.80(18)	109.67(12)	109.9(3)	110.1(2)
N12–C15–C16	110.22(17)	109.92(12)	110.1(3)	110.5(2)
C13–N11–C16	112.04(16)	111.99(12)	112.2(3)	112.6(2)
C15–N12–C14	111.13(17)	111.01(12)	111.5(3)	111.2(2)
N11–C13–C14–N12	–57.1(2)	–57.18(16)	–56.7(4)	–56.9(3)
N12–C15–C16–N11	55.4(2)	55.66(16)	54.6(4)	54.1(3)
C13–N11–C16–C15	–55.0(2)	–55.07(17)	–53.9(5)	–53.2(3)
C14–N12–C15–C16	–58.2(2)	–58.42(16)	–57.8(4)	–57.8(3)
C15–N12–C14–C13	59.1(2)	59.25(16)	58.9(5)	59.3(3)
C16–N11–C13–C14	55.9(2)	55.85(17)	55.0(4)	54.8(3)

Symmetry code: (I) $-x + 1, -y + 1, -z$.
